# Dr. George Woollam’s Case of Traumatic Tetanus, Successfully Treated by the Sesquicarbonate of Iron

**Published:** 1842-06-18

**Authors:** George Woollam


*Case of Traumatic Tetanus, successfully treated by the Sesquicarbonate of Iron.* By George Woollam, M. D.—W. B., aged 44, tall, muscular, of dark complexion, and rather irregular habits, a neighbouring farmer, in returning from market on March 29, 1840, fell, when a cart-wheel passed over his right hand. On the evening of the same day I was requested to visit him, and found the structures of the thumb much contused and lacerated. I continued to visit him until the 8th of April, when he came to my house to have the injured part dressed; and, as he was going on favourably, I saw no more of him until the 17th, at 9 A. M., when I was summoned to visit him; I found him lying on his left side, in a state, speaking in the language of the illustrious Cullen, of complete emprosthotonos, unable to turn, or even to move himself without assistance; deglutition was accomplished with extreme difficulty; the jaws were capable of being separated to such an extent as to admit of the introduction of a shallow spoon only between the teeth. The pulse was 108; pain in the epigastric region; a haggard appearance of countenance; a fixed prominent eye, with occasional profuse perspiration. On interrogating him regarding his absence from the 8th to the 17th, he replied, suffering excessive uneasiness from the mere effort of speaking, that he had been advised to betake himself to an old woman at Rotherham, who had treated him up to the latter date. On examining the wound, I found it covered with adhesive plaster, and in a very unfavorable condition; whereupon a bread poultice was substituted for the former dressing, six grains of calomel were given immediately, and, in a few hours after, two ounces of castor oil. Eight in the evening: No alleviation of the symptoms, although the bowels have been freely relieved; sesquicarbonate of iron, two drachms, to be taken every hour.

April 18. Nine, A. M. No alleviation. The iron to be continued, but in increased doses, and exhibited as frequently as possible. Eight, P. M.: Throat feels easier; no evacuation from the bowels since yesterday evening. The castor oil to be repeated as formerly.

19. Ten, A. M. Can swallow better; speak more distinctly; turn himself in the bed; and, when raised, can sit up, though in a bent position. Seven, P. M.: Met Dr. Shearman in consultation, who suggested a blister along the spine, and the exhibition of calomel and opium; but as there was a decided improvement, we agreed on continuing the iron, which was now exhibited to the extent of two ounces in two hours, and persevered in till the 27th, when the patient had so far recovered as to be able to walk abroad. From this period the dose was gradually diminished, and on the 11th of May entirely discontinued, from the disease having completely subsided.

In support of the utility of iron in neuralgic affections, it may be interesting to add, that a few weeks since Dr. Shearman, of Rotherham, had been consulted in a very formidable case of tetanic disease, which had supervened a fortnight previously to his being called. Dr. S., encouraged by the advantages which had been derived by my patient from the use of the iron, likewise exhibited it in the case to which he was called, and with the most perfect success.

The advantages which have resulted from the exhibition of iron in tetanic attacks ought to be promulgated, that the practice may attract the attention of such of our brethren as may be placed in situations and circumstances in which this formidable disease is more frequently met with than in this country. Sir James McGrigor observes, "All things have been fully tried in the army, in hundreds of cases, except iron." Dr. Elliotson employed iron in three instances, and in two of them successfully; in the third the symptoms were so violent that the patient died within twenty-four hours. The thirteenth volume of the "Medico-Chirurgical Transactions" contains reports of ten cases treated with iron, and of this number eight were successful. A previous knowledge of the history of my patient, and looking upon tetanus as a neuralgic affection, and the benefit which has often been known to result from the exhibition of this agent in tic douloureux, induced me to prescribe it in the foregoing case.—*Prov. Med. Journ.* April 30th, 1842.

